# Emerging neuromodulation treatments for opioid and stimulant use disorders

**DOI:** 10.3389/fnhum.2025.1570555

**Published:** 2025-10-14

**Authors:** Susanna D. Howard, Liming Qiu, Nathan Hager, Anna Rose Childress, Casey H. Halpern, Katherine W. Scangos

**Affiliations:** ^1^Department of Neurosurgery, University of Pennsylvania, Philadelphia, PA, United States; ^2^Department of Psychiatry, University of Pennsylvania, Philadelphia, PA, United States

**Keywords:** substance use disorder, opioid use disorder, stimulant use disorder, addiction, brain stimulation, neuromodulation

## Abstract

Over the past decade, deaths attributable to opioid and stimulant use have risen dramatically. While the U.S. Food and Drug Administration (FDA) has approved three medications for opioid use disorder, there is currently no FDA-approved treatment for stimulant use disorder. Despite the availability of medications for opioid use disorder, the rates of relapse and overdose, particularly in the time of widespread fentanyl use, remain distressingly high. There is an urgent need for more effective treatment options for these debilitating disorders. This article provides an overview of the current standard of care for opioid use disorder and stimulant use disorder. New and emerging neuromodulation approaches with a particular focus on deep brain stimulation are then discussed.

## 1 Introduction

Over 45 million people in the United States (U.S.) meet the Diagnostic and Statistical Manual of Mental Disorders, Fifth Edition (DSM-5) criteria for a substance use disorder (Portrait of American Healthcare, 2022). In the U.S., drug and alcohol use directly account for over 200,000 deaths annually (Ritchie et al., 2022). In 2019, 47,337 deaths in the U.S. were opioid-related–reflecting a 988% increase in opioid-related deaths since 1990 (Ritchie et al., 2022). While opioids have been responsible for the largest number of drug-related deaths, deaths associated with stimulant use have also risen dramatically in recent years. From 2012 to 2018, the rate of psychostimulant-related mortality increased by a factor of five (0.8–3.9 per 100,000 people) and cocaine-related mortality by a factor of three (1.4–4.5 per 100,000 people) (Ciccarone and Shoptaw, 2022). Increasingly, fentanyl has been intentionally introduced into the cocaine supply, (Di Trana et al., 2022) which has fueled a growth in co-occurring opioid and stimulant use and raised overdose rates (Ahmed et al., 2022).

The challenging treatment landscape compounds the devastating impacts of opioid use disorder (OUD) and stimulant use disorder (StUD). Buprenorphine, methadone, and naltrexone are U.S. Food and Drug Administration (FDA)-approved for the treatment of OUD; no existing treatments are FDA-approved for StUD (i.e., use of methamphetamine-type substances, cocaine, or misuse of prescription stimulants such as methylphenidate), causing significant impairment and distress. Furthermore, medications for substance use disorders are often difficult for patients to access conveniently and consistently (Cantor et al., 2021; Park et al., 2024). Neuromodulation techniques such as repetitive transcranial magnetic stimulation (rTMS), deep brain stimulation (DBS), and low-intensity focused ultrasound (LIFU) are promising new treatment modalities for substance use disorders that target dysfunctional neurocircuitry at the core of the disorders. In this mini-review, we will summarize the current standard of care for OUD and StUD. We will then discuss new and emerging neuromodulatory treatments for these two substance use disorders.

## 2 Standard of care for opioid and stimulant use disorders

Three medications - buprenorphine, methadone, and naltrexone - are FDA-approved and target the mu-opioid receptor with unique mechanisms of action (Leshner and Mancher, 2019). Methadone is a full agonist of the mu-opioid receptor with a long terminal half-life (up to 120 h) that reduces opioid craving, withdrawal, and stress reactivity. Methadone maintenance therapy has the highest treatment retention rates (Connery, 2015) and is highly effective (six randomized controlled trials, RR 0.66, 95% CI: 0.56 – 0.78) (Mattick et al., 2009). However, methadone has sedating effects and is difficult for many patients to access due to the burden of mandated daily in-person visits to the limited number of opioid treatment programs (Mitchell et al., 2022). Buprenorphine is a partial mu-opioid receptor agonist that competitively blocks or decreases the reinforcing effects of other opioids (Connery, 2015; Mattick et al., 2014). Unlike methadone, buprenorphine does not require daily in-person dispensing, and new formulations can extend dosing – even as far out as every 6-months (Soyka and Franke, 2021). However, it is less successful in retaining patients in treatment compared to methadone (Mattick et al., 2014). The adherence rates to naltrexone, a mu-opioid receptor antagonist that competitively blocks the effects of opioid agonists, are even lower, which has limited the real-world utility of this medication (Connery, 2015). This non-addictive treatment requires a period of abstinence before initiation and does not appear to directly reduce opioid craving (Dijkstra et al., 2007).

There is currently no FDA-approved treatment for StUD, highlighting an urgent gap in care. The growing need is driven by a significant increase in stimulant-related morbidity and mortality in recent years, which has even prompted the FDA to produce draft guidance to encourage drug development in this area (Center for Drug Evaluation and Research [CDER], 2023). A meta-analysis of behavioral and medical treatments for cocaine use disorder found that contingency management, a treatment plan that provides monetary rewards for negative drug tests, was the only treatment category associated with an increased likelihood of a negative urinalysis for cocaine (odds ratio 2.13, 95% CI 1.62 – 2.80) (Bentzley et al., 2021). However, in one of the largest trials of contingency management, the abstinence rate among participants remained less than 20%, Petry et al. (2005) and its real-world availability is limited (Bentzley et al., 2021; De Crescenzo et al., 2018). Off-label pharmacologic treatments for StUD include anticonvulsants, antidepressants, antipsychotics, dopamine agonists, opioids, and psychostimulants, but none significantly increase the odds of achieving negative urinalysis (Bentzley et al., 2021).

## 3 New and emerging neuromodulatory treatments

### 3.1 Non-invasive brain stimulation techniques

**FIGURE 1:**
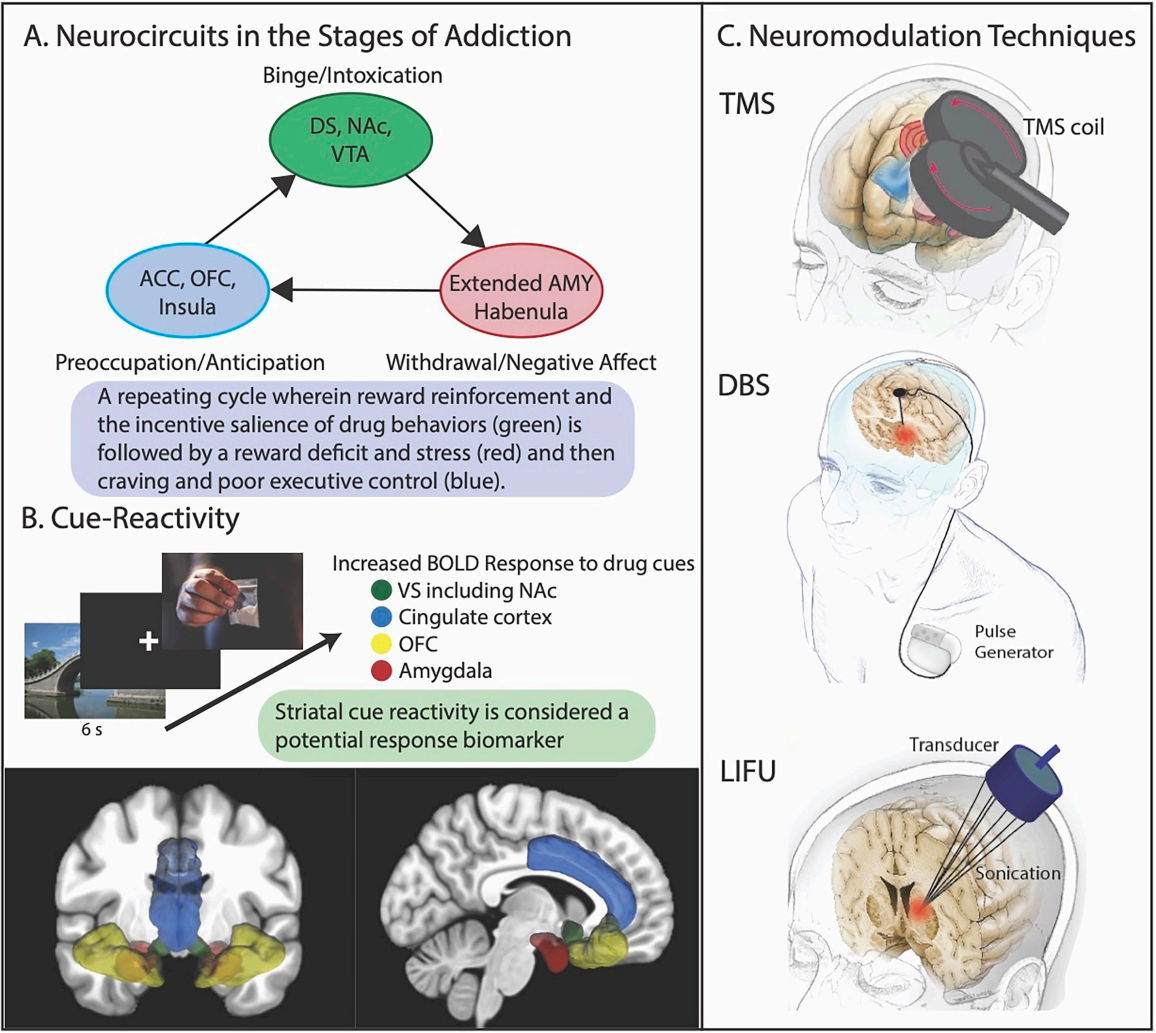
**(A)** Dysfunction within the mesocorticolimbic circuit, which processes rewards and punishments underlies substance use disorders. It is theorized that disturbances in three subcircuits underly the binge/intoxication, withdrawal/negative affect, and preoccupation/anticipation stages of addiction. **(B)** Brain reactivity to drug-related pictures during task-based fMRI studies. **(C)** The neuromodulation techniques available for treatment: transmagnetic stimulation (TMS), deep brain stimulation (DBS), and low-intensity focused ultrasound (LIFU). Abbreviations: amygdala (AMY), blood oxygen level dependent (BOLD), anterior cingulate cortex (ACC), nucleus accumbens (NAc), orbitofrontal cortex (OFC), ventral tegmental area (VTA).

Neuromodulation may fill the pressing unmet need for novel treatments by directly engaging dysfunctional circuitry (Figure 1) (Koob and Volkow, 2010, 2016). Table 1 includes studies of using non-invasive brain stimulation techniques for treatment of StUD and OUD. rTMS is a non-invasive method of neuromodulation that stimulates or inhibits neural activity by applying alternating magnetic fields to induce electric currents in underlying neurons in specific brain regions according to Faraday’s law of electromagnetic induction. It is FDA-approved for treatment-resistant depression, obsessive-compulsive disorder, and migraine with aura. rTMS has also been shown to be effective for smoking cessation, leading to FDA clearance of the BrainsWay Deep TMS system in 2020 (Zangen et al., 2021). Research into rTMS as a potential treatment for other substance use disorders is ongoing. The most commonly targeted region is the left dorsolateral prefrontal cortex (DLPFC), with varying stimulation parameters and treatment durations. High-frequency rTMS to the prefrontal cortex is hypothesized to reduce craving and drug cue reactivity and improve decision-making in the preoccupation/anticipation stage of addiction (Gorelick et al., 2014). Large sham-controlled double-blinded trials of left DLPFC rTMS have demonstrated decreased drug craving among subjects with StUD (Liang et al., 2018; Su et al., 2017, 2020a,2020b; Yuan et al., 2020). Left DLPFC rTMS has also been shown to be effective in reducing cue-induced craving in patients with OUD (Liu et al., 2020). A recent systematic review identified 18 studies including 985 patients [methamphetamine use disorder (*n* = 519), cocaine use disorder (*n* = 227), OUD (*n* = 239)] and showed mostly positive effects on cue-induced drug craving, though cocaine studies showed particularly mixed results (Mehta et al., 2024). The review highlighted several null or opposite effects of TMS on craving and found that little research has tested its effects on drug consumption. Statistically null results of some rTMS studies for substance use disorders could be the result of the type of coil employed (a figure-of-eight coil has a more focal stimulation field compared to an H-coil), inaccurate targeting, or strengthening of the sham/placebo effect with study visits and psychosocial support (Bolloni et al., 2016; Martinotti et al., 2022). Variable outcomes may also be attributable to suboptimal stimulation parameters and difficulty with retention in rTMS trials, which require daily treatments for up to 6 weeks (Brunoni et al., 2020). Advances in the delivery of neuromodulation may improve treatment efficacy and help address barriers to patient retention. Intermittent theta burst stimulation, a form of rTMS, has shortened treatment times while maintaining efficacy (Liu et al., 2022). In one of the largest rTMS studies for StUD, 126 participants with methamphetamine disorder were randomized to either 20 daily sessions of intermittent theta burst stimulation to the DLPFC or sham treatment (Su et al., 2020a). The theta burst stimulation group experienced a significant decline in cue-induced craving which was not observed in the sham group. This study could not assess the effect of theta burst stimulation on abstinence as it took place in a long-term residential treatment facility. Accelerated TMS paradigms, where the full course of TMS is compressed into 5 days, have shown efficacy for depression (Cole et al., 2022) and may hold promise for substance use disorders. Such treatments could also be performed inpatient, which increases the likelihood of TMS course completion. One such study, evaluating the feasibility of accelerated rTMS for StUD and comorbid depression, is currently underway (NCT06424184). In addition to reducing craving, the effect of TMS on drug use abstinence and relapse is an important area for further study.

**TABLE 1:** Studies of non-invasive brain stimulation techniques [repetitive transcranial magnetic stimulation (rTMS), transcranial direct current stimulation (tDCS), transcranial alternating current stimulation (tACS)], deep brain stimulation (DBS) and low intensity focused ultrasound (LIFU) for stimulant use disorder and opioid use disorder.

Study	Drug of use	Intervention	Sample size	Results	Adverse events
**Non-invasive brain stimulation techniques**					
Batista et al., 2015	Crack-cocaine	Five sessions of bilateral DLPFC tDCS (left cathodal, right anodal)	17	Craving scores were significantly reduced in the treatment group compared to sham.	Mild side effects including altered scalp sensation (buzzing, tingling, burning).
Wang et al., 2016	Heroin	Single tDCS session to the bilateral frontal-parietal-temporal areas.	20	Cue-induced craving scores were significantly reduced in the treatment group whereas there was no changes in craving in the control group.	No side effects reported.
Yuan et al., 2020	Heroin	10-Hz and 1-Hz rTMS to the left DLPFC	118	Cue-induced craving was significantly reduced in treatment group compared to control group receiving no rTMS treatment.	Mild side effects including dizziness, headache, neck pain, insomnia.
Liu et al., 2020	Methamphetamine	1-Hz rTMS to the left prefrontal cortex	73	Impulse inhibition was significantly improved in the treatment group as well as reduced self-reported cue-induced craving compared to the sham group.	None reported.
**Deep brain stimulation**					
Zhou et al., 2011	Heroin	Continuous bilateral NAc DBS.	1	No relapse at 6-year follow-up. Improvement in memory scores, and reduction in depression and anxiety measures.	Mild confusion, urinary incontinence, weight gain.
Valencia-Alfonso et al., 2012	Heroin	Continuous bilateral NAC/ALIC DBS.	1	Six months without heroin use with exception of a 14-day relapse.	None reported.
Kuhn et al., 2014	Opioids (& methamphetamine)	Continuous bilateral NAc DBS.	2	Continuous abstinence with exception of one singular incident of heroin consumption for both patients. Follow-up time not reported.	Seizure in patient with prior history of seizures.
Gonçalves-Ferreira et al., 2016	Cocaine	Continuous bilateral ALIC/nucleus stria terminalis/NAc DBS. Three-phase crossover design.	1	At 24-month follow-up, 68% weeks free of consumption vs. 41% before and 56.5% negative urinalysis vs. 12% before.	Transient stimulation side effects.
Ge et al., 2019	Methamphetamine	Continuous bilateral NAc/ALIC DBS.	2	One participant without relapse at 1.5-year follow-up. Minimal effect in one participant who started intermittently relapsing at 6-month follow-up.	Transient hypomania, anxiety.
Zhang et al., 2019	Methamphetamine	Continuous bilateral NAc/ventral capsule DBS.	1	Abstinence for a full year.	None reported.
Rezai et al., 2024	Opioids (& benzodiazepines, cannabis)	Continuous bilateral NAc/ventral capsule DBS.	4	Two participants with complete abstinence. One participant with reduced frequency and severity of drug use. One participant had device explanted due to non-compliance with treatment requirements.	No serious adverse events or device- or stimulation-related adverse events.
**Focused ultrasound**					
Mahoney et al., 2023	Opioids	Two doses (60 and 90 W) of LIFU to bilateral NAc.	4	Two participants receiving 90 W LIFU dose had decreased craving 90 days following treatment. Urine toxicology was negative for opioids through 90-day follow-up for all four participants.	No serious adverse events.
Rezai et al., 2025	Opioid (& stimulants)	One dose (90–100 W) of LIFU to bilateral NAc	8	Five participants were abstinent at 90-day follow-up. The three participants who experienced relapse used drugs less frequently than their baseline use.	No serious adverse events.

ALIC, anterior limb of internal capsule; DLPFC, dorsolateral prefrontal cortex; NAc, nucleus accumbens; W, Watt.

Other promising non-invasive brain stimulation techniques include transcranial direct current stimulation (tDCS) and transcranial alternating current stimulation (tACS). tDCS modulates cortical excitability via non-synaptic changes of the cells–including cathodal stimulation to decrease cortical excitability and anodal stimulation to increase cortical excitability via either hyperpolarization or depolarization, respectively, of the resting membrane potential (Stagg and Nitsche, 2011). While tDCS constantly depolarizes or hyperpolarizes neurons, tACS delivers fluctuating current between electrodes to induce synaptic plasticity (Elyamany et al., 2021; Gholamali Nezhad et al., 2024). In comparison to rTMS, tDCS and tACS do not require a magnetic coil and are delivered by relatively inexpensive, portable battery-powered devices (Elder and Taylor, 2014). This increases the accessibility of these modalities, however, unlike rTMS, tDCS and tACS are not able to focally deliver stimulation to specific targets. tDCS treatments (most commonly directed to the DLPFC) have been shown to reduce craving for stimulants [cocaine, (Batista et al., 2015; Klauss et al., 2018) methamphetamine (Shahbabaie et al., 2014; Shariatirad et al., 2016)] and heroin (Wang et al., 2016). tACS is a newer technology than tDCS and only two studies have tested it for substance use disorder indications. These studies applied alpha-tACS to the bilateral DLPFC, with one showing improved inhibitory control in people with a variety of substance use disorders (*N* = 30) (Daughters et al., 2020) and the other showing improved behavioral flexibility in people with prior substance use dependence (*N* = 17) (McKim et al., 2021). Research has yet to test the effects of tACS on OUD and StUDs or on drug use and craving.

### 3.2 Deep brain stimulation (DBS)

One limitation of currently available non-invasive brain stimulation techniques is the inability to directly target the subcortical brain structures involved in the reward and reinforcement circuit. DBS and LIFU can successfully reach deeper brain targets. Table 1 lists the outcomes of all reported studies of DBS and LIFU for the treatment of OUD and StUDs.

In DBS, electrical current is delivered to a deep brain region via implanted electrodes. It is a standard-of-care treatment for advanced movement disorders such as essential tremor and Parkinson’s disease, and epilepsy with over 5,500 DBS operations performed each year in the U.S (Sarica et al., 2023). The electrodes are connected via extension wires to a pacemaker-like unit called an implantable pulse generator, which is typically placed subcutaneously in the chest wall. There are several proposed non-exclusive therapeutic mechanisms of DBS. One predominant theory is that the benefits of high-frequency [>130 Hertz (Hz)] stimulation arise from a reversible lesioning effect (Herrington et al., 2016). There is also increasing recognition that DBS modulates neural activity on a network level to effect dysregulated connectivity–the electrode target acting as a single node with upstream and downstream projections (Hollunder et al., 2024). Like TMS, DBS treatment involves multiple programming parameters (frequency, current amplitude, pulse width) that can be adjusted to maximize clinical efficacy and minimize side effects.

Pre-clinical studies have shown that nucleus accumbens (NAc) DBS reduces drug-seeking behavior in rodents (Eskandari et al., 2023; Guercio et al., 2015; Guo et al., 2013). Reinstatement testing, commonly used in animal studies of addiction, is designed to model relapse. After a period of drug self-administration followed by extinction training, drug-associated cues are re-introduced to test for drug-seeking behavior. Using this paradigm, Vassoler et al. (2008) demonstrated that DBS of the NAc shell reduced drug seeking in rats. Rats who received bilateral NAc DBS had lower the number of lever presses to trigger cocaine administration following reinstatement, suggesting attenuation of drug-seeking behavior with stimulation.

Several human studies in a small number of patients have demonstrated the feasibility of continuous NAc DBS for StUDs and OUDs (Table 1). Most studies showed that participants remained abstinent or reduced the amount and/or frequency of drug use. Across these studies, no serious adverse events were reported. Zhang et al. (2019) reported the results of bilateral NAc DBS in a patient with treatment-refractory methamphetamine disorder. Following 1 year of DBS treatment, the patient remained abstinent per self-report and urine and hair drug testing. The patient also had improvements in measures of craving, mood, anxiety, quality of life, and functional impairment. Positron emission tomography imaging obtained before and 1-year post-DBS implantation showed enhanced striatal dopamine transporter binding, suggesting a normalization of brain dopaminergic dysfunction. Rezai et al. (2024) conducted a prospective, open-label, single-arm study of bilateral NAc DBS in treatment-refractory OUDs. Two of the four patients were completely abstinent after surgery, one patient had recurrent drug use but decreased frequency and severity, and one patient had the DBS system explanted due to non-compliance with study protocols. In light of the promising early findings, several groups are now designing randomized-controlled trials to establish the safety and feasibility of NAc DBS for substance use disorder (NCT04354077).

DBS provides continuous treatment with a less demanding follow-up schedule than methadone maintenance therapy. However, frequent programming visits to optimize stimulation settings can be a barrier (Hunka et al., 2005). In 2020, the first fully remote DBS programming system received FDA approval, which may substantially improve the feasibility of this treatment option (Merola et al., 2021). Another recent advance is the commercial availability of devices with the capability to continuously record local field potentials, i.e., synaptic potentials of adjacent neurons at the implantation site. The ability to detect behaviorally relevant brain signals brings promise to closed-loop delivery of DBS only when needed (i.e., when a pathological signal is detected). Electrophysiological biomarkers of reward anticipation or craving have been identified in animals (Wu et al., 2018) and humans (Nho et al., 2023) through the use of intracranial recordings and may potentially be integrated into new DBS technology (Chen et al., 2024). In closed-loop DBS, stimulation is delivered only when a biomarker is detected (responsive DBS), or stimulation is adjusted based on the presence or magnitude of a biomarker (adaptive DBS). In a patient with a history of OUD and StUD, we identified a drug-cue specific low-frequency (1–7 Hz) band electrophysiological signal in the left NAc shell (Qiu et al., 2024). This biomarker was not identified in other electrode contacts nor with other behavioral tasks. Stimulation in the NAc shell attenuated the power of this low-frequency band signal as well as clinical ratings of craving. This suggests the potential of using this low-frequency signal as a biomarker of craving, and ability to modulate this signal, and thus behavior, with DBS. The feasibility of closed-loop stimulation has been demonstrated in Parkinson’s disease, epilepsy, and some neuropsychiatric conditions (Oehrn et al., 2024). Like the symptoms of Parkinson’s disease, the urges and cravings associated with substance use disorder are often dynamic and context-dependent. Closed-loop capabilities can permit the identification and modulation of urge/craving states in real time. This flexibility would enable treatment of both background affective symptoms in addition to cue-reactive states to further enhance recovery. Other advantages of closed-loop stimulation include decreasing the total stimulation burden, which may reduce side effects, minimize tachyphylaxis phenomenon, improve outcomes, and facilitate neural plasticity.

### 3.3 Focused ultrasound

Low-intensity focused ultrasound is an exciting and new neuromodulation technique that can reach deep structures but, unlike DBS, does not require open neurosurgery or a device implant (Olaitan et al., 2024). While high-intensity focused ultrasound uses thermal energy to ablate surgical targets, LIFU is non-ablative and is thought to modulate neuronal activity through mechanotransduction effects and can excite or inhibit neuronal activity depending on the specific parameters. While still nascent in its development, a pilot open-label study including four participants with OUD who received LIFU [either 60 or 90 Watt (W) dose] to the bilateral NAc demonstrated safety and promising outcomes on craving (Mahoney et al., 2023). The two participants receiving the enhanced LIFU dose (90 W) had decreased craving for substances acutely and at 90 days following treatment. In a follow-up study (NCT04197921) of a similar but larger sample (*N* = 8), investigators again found reductions in cue-induced craving following 90–100 W sonication to the NAc and further showed sustained decreases in substance use (Rezai et al., 2025). Building on these results, there are currently five active LIFU studies for substance use disorder indications, including another study investigating the safety and tolerability of LIFU for OUD (NCT06218706) and one study seeking to understand the impact of LIFU on craving levels among patients with cocaine use disorder as evidenced by imaging of the dorsal anterior insula and subjective ratings (NCT05857852). This reflects the strong interests and growth of LIFU as a potential neuromodulatory therapy in substance use disorders.

## 4 Discussion

The current standard of care for patients with substance use disorders is severely lacking, leaving many patients refractory to existing therapies. The resulting morbidity and mortality are substantial and represent an unacceptable *status quo*. The dysfunctional neurocircuitry driving addiction is theoretically amenable to neuromodulation as a treatment, and the results from cases of neuromodulation in humans with severe, refractory substance use disorders are promising. However, the nature of this disorder challenges retention rates in complex treatment trials, which, together with a lack of consensus and standardization of reliable outcome measures and biomarkers, has hindered the necessary clinical development pathway to FDA approval.

Growing efforts seek to standardize the reduction in substance use rather than abstinence as a primary outcome measure. FDA draft guidance released in October 2023 highlights within-subject change in the pattern of drug use as an appropriate clinical trial endpoint (Center for Drug Evaluation and Research [CDER], 2023). Yet, the lack of reliability of self-report measures and the burden of frequent urine drug screens still remain as challenges. The use of multiple substances further complicates the outcomes for specific substances. As OUD and StUDs increasingly co-occur and involve overlapping neurocircuitry, there is a scientific rationale for neuromodulation trial designs that encompass both conditions. fMRI-cue reactivity is an exciting potential surrogate endpoint that is widely accepted by researchers in the field (Ekhtiari et al., 2022). Cue reactivity is a learned response to drug cues that triggers prefrontal cortex-driven craving along with conditioned activation of the subcortical motivational (reward) circuitry. Objective neuroimaging biomarkers of cue reactivity may help identify people at risk for recurrence and measure the progression and treatment of the disorder (Childress et al., 2008; Ekhtiari et al., 2024; Morales and Berridge, 2020). fMRI studies across substance use disorders consistently identify increased activity in the striatum, amygdala, PFC, and insula in response to drug cues, (Dejoie et al., 2023; Hill-Bowen et al., 2021; Huang et al., 2018; Moeller and Paulus, 2018; Regier et al., 2021; Yalachkov et al., 2012) which is associated with substance use severity, treatment outcomes, and risk of relapse (Janes et al., 2010; Jasinska et al., 2014; Li et al., 2015; Moeller and Paulus, 2018). While fMRI has high spatial resolution, the temporal resolution is poor compared to electroencephalography (EEG) (Houston and Schlienz, 2018). Event-related potentials (ERP), EEG brain signals measured in response to a specific stimulus, have been associated with treatment outcomes, relapse, and cue reactivity in patients with substance use disorders (Bel-Bahar et al., 2022; Habelt et al., 2020; Parvaz et al., 2017; Sokhadze and Shaban, 2022) EEG biomarkers may serve as a more practical and temporally dynamic endpoint compared to fMRI biomarkers. Current efforts to standardize experimental design and analysis of these markers are laudable and may lead to the necessary extensive validation before such markers (neuroimaging or signal-based) can support regulatory approval (Ekhtiari et al., 2022, 2024).

Future designs of randomized controlled studies also need to account for key practical considerations relevant to the care of patients with substance use disorder to ensure the feasibility of these interventions on a larger scale outside of an investigative context. Novel approaches to neurostimulation, including accelerated TMS and LIFU, may help achieve this goal by reducing total intervention time and more directly targeting the reward circuitry underlying craving and addiction. Ongoing refinement of neuromodulatory techniques and advancement in the understanding of neurocircuitry will further improve their efficacy and may transform the treatment landscape for substance use disorders. While the NAc for DBS and focused ultrasound and the DLPFC for non-invasive stimulation techniques have been the predominant targets for addiction treatment, the optimal target for each modality is not fully established and an area of ongoing research (Mehta et al., 2024; Zammit Dimech et al., 2024). Neuromodulation treatment will likely be concentrated in tertiary, academic medical centers at first. Therefore, it will be critical to target outreach to communities with decreased healthcare access.

## References

[B1] Ahmed S. Sarfraz Z. Sarfraz A. (2022). Editorial: A changing epidemic and the rise of opioid-stimulant co-use. *Front. Psychiatry* 13:918197. 10.3389/fpsyt.2022.918197 35873238 PMC9296817

[B2] Batista E. K. Klauss J. Fregni F. Nitsche M. A. Nakamura-Palacios E. M. A. (2015). Randomized placebo-controlled trial of targeted prefrontal cortex modulation with bilateral tDCS in patients with crack-cocaine dependence. *Int. J. Neuropsychopharmacol.* 18:yv066. 10.1093/ijnp/pyv066 26065432 PMC4675977

[B3] Bel-Bahar T. S. Khan A. A. Shaik R. B. Parvaz M. A. (2022). A scoping review of electroencephalographic (EEG) markers for tracking neurophysiological changes and predicting outcomes in substance use disorder treatment. *Front. Hum. Neurosci.* 16:995534. 10.3389/fnhum.2022.995534 36325430 PMC9619053

[B4] Bentzley B. S. Han S. S. Neuner S. Humphreys K. Kampman K. M. Halpern C. H. (2021). comparison of treatments for cocaine use disorder among adults: A systematic review and meta-analysis. *JAMA Netw. Open* 4:e218049. 10.1001/jamanetworkopen.2021.8049 33961037 PMC8105751

[B5] Bolloni C. Panella R. Pedetti M. Frascella A. G. Gambelunghe C. Piccoli T. (2016). Bilateral transcranial magnetic stimulation of the prefrontal cortex reduces cocaine intake: A pilot study. *Front. Psychiatry* 7:133. 10.3389/fpsyt.2016.00133 27551268 PMC4976094

[B6] Brunoni A. R. Arns M. Baeken C. Blumberger D. Brunelin J. Carpenter L. L. (2020). Mixing apples and oranges in assessing outcomes of repetitive transcranial stimulation meta-analyses. *Psychother. Psychosom.* 89 106–107. 10.1159/000504653 31794971

[B7] Cantor J. Powell D. Kofner A. Stein B. D. (2021). Population-based estimates of geographic accessibility of medication for opioid use disorder by substance use disorder treatment facilities from 2014 to 2020. *Drug Alcohol. Depend.* 229(Pt A), 109107. 10.1016/j.drugalcdep.2021.109107 34656034 PMC8981564

[B8] Center for Drug Evaluation and Research [CDER] (2023). *Stimulant use disorders: Developing drugs for treatment.* Available online at: https://www.fda.gov/regulatory-information/search-fda-guidance-documents/stimulant-use-disorders-developing-drugs-treatment (accessed October 16, 2024).

[B9] Chen D. Zhao Z. Shi J. Li S. Xu X. Wu Z. (2024). Harnessing the sensing and stimulation function of deep brain-machine interfaces: A new dawn for overcoming substance use disorders. *Transl. Psychiatry* 14:440. 10.1038/s41398-024-03156-8 39419976 PMC11487193

[B10] Childress A. R. Ehrman R. N. Wang Z. Li Y. Sciortino N. Hakun J. (2008). Prelude to passion: Limbic activation by “unseen” drug and sexual cues. *PLoS One* 3:e1506. 10.1371/journal.pone.0001506 18231593 PMC2204052

[B11] Ciccarone D. Shoptaw S. (2022). Understanding stimulant use and use disorders in a new era. *Med. Clin. North Am.* 106 81–97. 10.1016/j.mcna.2021.08.010 34823736 PMC8670631

[B12] Cole E. J. Phillips A. L. Bentzley B. S. Stimpson K. H. Nejad R. Barmak F. (2022). Stanford Neuromodulation Therapy (SNT): A double-blind randomized controlled trial. *Am. J. Psychiatry* 179 132–141. 10.1176/appi.ajp.2021.20101429 34711062

[B13] Connery H. S. (2015). Medication-assisted treatment of opioid use disorder: Review of the evidence and future directions. *Harv. Rev. Psychiatry* 23 63–75. 10.1097/HRP.0000000000000075 25747920

[B14] Daughters S. B. Yi J. Y. Phillips R. D. Carelli R. M. Fröhlich F. (2020). Alpha-tACS effect on inhibitory control and feasibility of administration in community outpatient substance use treatment. *Drug Alcohol. Depend.* 213:108132. 10.1016/j.drugalcdep.2020.108132 32593154 PMC8815795

[B15] De Crescenzo F. Ciabattini M. D’Alò G. L. De Giorgi R. Del Giovane C. Cassar C. (2018). Comparative efficacy and acceptability of psychosocial interventions for individuals with cocaine and amphetamine addiction: A systematic review and network meta-analysis. *PLoS Med.* 15:e1002715. 10.1371/journal.pmed.1002715 30586362 PMC6306153

[B16] Dejoie J. M. Senia N. Konova A. Smith D. Fareri D. (2023). Common and distinct drug cue reactivity patterns associated with cocaine and heroin: An fMRI meta-analysis. *medRxiv [Preprint]* 10.1101/2023.10.19.23297268 40800429 PMC12272201

[B17] Di Trana A. Berardinelli D. Montanari E. Berretta P. Basile G. Huestis M. A. (2022). Molecular insights and clinical outcomes of drugs of abuse adulteration: New trends and new psychoactive substances. *Int. J. Mol. Sci.* 23:14619. 10.3390/ijms232314619 36498947 PMC9739917

[B18] Dijkstra B. A. De Jong C. A. Bluschke S. M. Krabbe P. F. van der Staak C. P. (2007). Does naltrexone affect craving in abstinent opioid-dependent patients? *Addict. Biol.* 12 176–182. 10.1111/j.1369-1600.2007.00067.x 17508990

[B19] Ekhtiari H. Sangchooli A. Carmichael O. Moeller F. G. O’Donnell P. Oquendo M. (2024). Neuroimaging biomarkers in addiction. *medRxiv [Preprint]* 10.1101/2024.09.02.24312084 39281741 PMC11398440

[B20] Ekhtiari H. Zare-Bidoky M. Sangchooli A. Janes A. C. Kaufman M. J. Oliver J. A. (2022). A methodological checklist for fMRI drug cue reactivity studies: Development and expert consensus. *Nat. Protoc.* 17 567–595. 10.1038/s41596-021-00649-4 35121856 PMC9063851

[B21] Elder G. J. Taylor J. P. (2014). Transcranial magnetic stimulation and transcranial direct current stimulation: Treatments for cognitive and neuropsychiatric symptoms in the neurodegenerative dementias? *Alzheimers Res. Ther.* 6:74. 10.1186/s13195-014-0074-1 25478032 PMC4255638

[B22] Elyamany O. Leicht G. Herrmann C. S. Mulert C. (2021). Transcranial alternating current stimulation (tACS): From basic mechanisms towards first applications in psychiatry. *Eur. Arch. Psychiatry Clin. Neurosci.* 271 135–156. 10.1007/s00406-020-01209-9 33211157 PMC7867505

[B23] Eskandari K. Fattahi M. Yazdanian H. Haghparast A. (2023). Is deep brain stimulation an effective treatment for psychostimulant dependency? a preclinical and clinical systematic review. *Neurochem. Res.* 48 1255–1268 10.1007/s11064-022-03818-3 36445490

[B24] Ge S. Chen Y. Li N. Qu L. Li Y. Jing J. (2019). Deep brain stimulation of nucleus accumbens for methamphetamine addiction: Two case reports. *World Neurosurg.* 122 512–517. 10.1016/j.wneu.2018.11.056 30448569

[B25] Gholamali Nezhad F. Martin J. Tassone V. K. Swiderski A. Demchenko I. Khan S. (2024). Transcranial alternating current stimulation for neuropsychiatric disorders: A systematic review of treatment parameters and outcomes. *Front. Psychiatry* 15:1419243. 10.3389/fpsyt.2024.1419243 39211537 PMC11360874

[B26] Gonçalves-Ferreira A. do Couto F. S. Rainha Campos A. Lucas Neto L. P. Gonçalves-Ferreira D. Teixeira J. (2016). Deep brain stimulation for refractory cocaine dependence. *Biol. Psychiatry* 79 e87–e89. 10.1016/j.biopsych.2015.06.023 26235303

[B27] Gorelick D. A. Zangen A. George M. S. (2014). Transcranial magnetic stimulation in the treatment of substance addiction. *Ann. N. Y. Acad. Sci.* 1327 79–93. 10.1111/nyas.12479 25069523 PMC4206564

[B28] Guercio L. A. Schmidt H. D. Pierce R. C. (2015). Deep brain stimulation of the nucleus accumbens shell attenuates cue-induced reinstatement of both cocaine and sucrose seeking in rats. *Behav. Brain Res.* 281 125–130. 10.1016/j.bbr.2014.12.025 25529183 PMC4309556

[B29] Guo L. Zhou H. Wang R. Xu J. Zhou W. Zhang F. (2013). DBS of nucleus accumbens on heroin seeking behaviors in self-administering rats. *Drug Alcohol Depend.* 129 70–81. 10.1016/j.drugalcdep.2012.09.012 23062870

[B30] Habelt B. Arvaneh M. Bernhardt N. Minev I. (2020). Biomarkers and neuromodulation techniques in substance use disorders. *Bioelectron. Med.* 6:4. 10.1186/s42234-020-0040-0 32232112 PMC7098236

[B31] Herrington T. M. Cheng J. J. Eskandar E. N. (2016). Mechanisms of deep brain stimulation. *J. Neurophysiol.* 115 19–38. 10.1152/jn.00281.2015 26510756 PMC4760496

[B32] Hill-Bowen L. D. Riedel M. C. Poudel R. Salo T. Flannery J. S. Camilleri J. A. (2021). The cue-reactivity paradigm: An ensemble of networks driving attention and cognition when viewing drug and natural reward-related stimuli. *Neurosci. Biobehav. Rev.* 130 201–213. 10.1016/j.neubiorev.2021.08.010 34400176 PMC8511211

[B33] Hollunder B. Ostrem J. L. Sahin I. A. Rajamani N. Oxenford S. Butenko K. (2024). Mapping dysfunctional circuits in the frontal cortex using deep brain stimulation. *Nat. Neurosci.* 27 573–586. 10.1038/s41593-024-01570-1 38388734 PMC10917675

[B34] Houston R. J. Schlienz N. J. (2018). Event-Related potentials as biomarkers of behavior change mechanisms in substance use disorder treatment. *Biol. Psychiatry Cogn. Neurosci. Neuroimag.* 3 30–40. 10.1016/j.bpsc.2017.09.006 29397076 PMC5801766

[B35] Huang Y. Mohan A. De Ridder D. Sunaert S. Vanneste S. (2018). The neural correlates of the unified percept of alcohol-related craving: A fMRI and EEG study. *Sci. Rep.* 8:923. 10.1038/s41598-017-18471-y 29343732 PMC5772563

[B36] Hunka K. Suchowersky O. Wood S. Derwent L. Kiss Z. H. (2005). Nursing time to program and assess deep brain stimulators in movement disorder patients. *J. Neurosci. Nurs.* 37 204–210. 10.1097/01376517-200508000-00006 16206546

[B37] Janes A. C. Pizzagalli D. A. Richardt S. deB Frederick B. Chuzi S. Pachas G. (2010). Brain reactivity to smoking cues prior to smoking cessation predicts ability to maintain tobacco abstinence. *Biol. Psychiatry* 67 722–729. 10.1016/j.biopsych.2009.12.034 20172508 PMC2954596

[B38] Jasinska A. J. Stein E. A. Kaiser J. Naumer M. J. Yalachkov Y. (2014). Factors modulating neural reactivity to drug cues in addiction: A survey of human neuroimaging studies. *Neurosci. Biobehav. Rev.* 38 1–16. 10.1016/j.neubiorev.2013.10.013 24211373 PMC3913480

[B39] Klauss J. Anders Q. S. Felippe L. V. Ferreira L. V. B. Cruz M. A. Nitsche M. A. (2018). Lack of effects of extended sessions of transcranial direct current stimulation (tDCS) over dorsolateral prefrontal cortex on craving and relapses in crack-cocaine users. *Front. Pharmacol.* 9:1198. 10.3389/fphar.2018.01198 30405414 PMC6206545

[B40] Koob G. F. Volkow N. D. (2010). Neurocircuitry of addiction. *Neuropsychopharmacology* 35 217–238. 10.1038/npp.2009.110 19710631 PMC2805560

[B41] Koob G. F. Volkow N. D. (2016). Neurobiology of addiction: A neurocircuitry analysis. *Lancet Psychiatry* 3 760–773. 10.1016/S2215-0366(16)00104-8 27475769 PMC6135092

[B42] Kuhn J. Möller M. Treppmann J. F. Bartsch C. Lenartz D. Gruendler T. O. (2014). Deep brain stimulation of the nucleus accumbens and its usefulness in severe opioid addiction. *Mol. Psychiatry* 19 145–146. 10.1038/mp.2012.196 23337942

[B43] Leshner A. I. Mancher M. (eds) (2019). *Medications for opioid use disorder save lives.* Washington, DC: National Academies Press, 10.17226/25310 30896911

[B44] Li Q. Li W. Wang H. Wang Y. Zhang Y. Zhu J. (2015). Predicting subsequent relapse by drug-related cue-induced brain activation in heroin addiction: An event-related functional magnetic resonance imaging study. *Addict. Biol.* 20 968–978. 10.1111/adb.12182 25214465

[B45] Liang Y. Wang L. Yuan T. F. (2018). Targeting withdrawal symptoms in men addicted to methamphetamine with transcranial magnetic stimulation: A randomized clinical trial. *JAMA Psychiatry* 75 1199–1201. 10.1001/jamapsychiatry.2018.2383 30208372 PMC6583874

[B46] Liu Q. Sun H. Hu Y. Wang Q. Zhao Z. Dong D. (2022). Intermittent theta burst stimulation vs. high-frequency repetitive transcranial magnetic stimulation in the treatment of methamphetamine patients. *Front. Psychiatry* 13:842947. 10.3389/fpsyt.2022.842947 35558419 PMC9087275

[B47] Liu X. Zhao X. Liu T. Liu Q. Tang L. Zhang H. (2020). The effects of repetitive transcranial magnetic stimulation on cue-induced craving in male patients with heroin use disorder. *EBioMedicine* 56:102809. 10.1016/j.ebiom.2020.102809 32512513 PMC7276507

[B48] Mahoney J. J. Haut M. W. Carpenter J. Ranjan M. Thompson-Lake D. G. Y. Marton J. L. (2023). Low-intensity focused ultrasound targeting the nucleus accumbens as a potential treatment for substance use disorder: Safety and feasibility clinical trial. *Front. Psychiatry* 14:1211566. 10.3389/fpsyt.2023.1211566 37779628 PMC10540197

[B49] Martinotti G. Pettorruso M. Montemitro C. Spagnolo P. A. Acuti Martellucci C. Di Carlo F. (2022). Repetitive transcranial magnetic stimulation in treatment-seeking subjects with cocaine use disorder: A randomized, double-blind, sham-controlled trial. *Prog. Neuropsychopharmacol. Biol. Psychiatry* 116:110513. 10.1016/j.pnpbp.2022.110513 35074451

[B50] Mattick R. P. Breen C. Kimber J. Davoli M. (2009). Methadone maintenance therapy versus no opioid replacement therapy for opioid dependence. *Cochrane Database Syst. Rev.* 2009:CD002209. 10.1002/14651858.CD002209.pub2 12519570

[B51] Mattick R. P. Breen C. Kimber J. Davoli M. (2014). *Buprenorphine maintenance versus placebo or methadone maintenance for opioid dependence - Mattick, RP.* London: Cochrane Library. 10.1002/14651858.CD002207.pub3 18425880

[B52] McKim T. H. Dove S. J. Robinson D. L. Fröhlich F. Boettiger C. A. (2021). Addiction history moderates the effect of prefrontal 10-Hz transcranial alternating current stimulation on habitual action selection. *J. Neurophysiol.* 125 768–780. 10.1152/jn.00180.2020 33356905 PMC7988748

[B53] Mehta D. D. Praecht A. Ward H. B. Sanches M. Sorkhou M. Tang V. M. (2024). A systematic review and meta-analysis of neuromodulation therapies for substance use disorders. *Neuropsychopharmacology* 49 649–680. 10.1038/s41386-023-01776-0 38086901 PMC10876556

[B54] Merola A. Singh J. Reeves K. Changizi B. Goetz S. Rossi L. (2021). New frontiers for deep brain stimulation: Directionality, sensing technologies, remote programming, robotic stereotactic assistance, asleep procedures, and connectomics. *Front. Neurol.* 12:694747. 10.3389/fneur.2021.694747 34367055 PMC8340024

[B55] Mitchell P. Samsel S. Curtin K. M. Price A. Turner D. Tramp R. (2022). Geographic disparities in access to medication for opioid use disorder across us census tracts based on treatment utilization behavior. *Soc. Sci. Med.* 302:114992. 10.1016/j.socscimed.2022.114992 35512612

[B56] Moeller S. J. Paulus M. P. (2018). Toward biomarkers of the addicted human brain: Using neuroimaging to predict relapse and sustained abstinence in substance use disorder. *Prog. Neuropsychopharmacol. Biol. Psychiatry* 80(Pt B), 143–154. 10.1016/j.pnpbp.2017.03.003 28322982 PMC5603350

[B57] Morales I. Berridge K. C. (2020). ‘Liking’ and ‘wanting’ in eating and food reward: Brain mechanisms and clinical implications. *Physiol. Behav.* 227:113152. 10.1016/j.physbeh.2020.113152 32846152 PMC7655589

[B58] Nho Y. H. Rolle C. E. Topalovic U. Shivacharan R. S. Cunningham T. N. Hiller S. (2023). Responsive deep brain stimulation guided by ventral striatal electrophysiology of obsession durably ameliorates compulsion. *Neuron* 112 73–83.e4. 10.1016/j.neuron.2023.09.034. 37865084 PMC10841397

[B59] Oehrn C. R. Cernera S. Hammer L. H. Shcherbakova M. Yao J. Hahn A. (2024). Chronic adaptive deep brain stimulation versus conventional stimulation in Parkinson’s disease: A blinded randomized feasibility trial. *Nat. Med.* 30 3345–3356. 10.1038/s41591-024-03196-z 39160351 PMC11826929

[B60] Olaitan G. O. Lynch W. J. Venton B. J. (2024). The therapeutic potential of low-intensity focused ultrasound for treating substance use disorder. *Front. Psychiatry* 15:1466506. 10.3389/fpsyt.2024.1466506 39628494 PMC11612502

[B61] Park T. W. Shuey B. Liebschutz J. Cantor J. Anderson T. S. (2024). Treatment approaches for opioid use disorder offered in US substance use treatment facilities. *JAMA* 332 502–504. 10.1001/jama.2024.11913 38990551 PMC11240225

[B62] Parvaz M. A. Moeller S. J. Malaker P. Sinha R. Alia-Klein N. Goldstein R. Z. (2017). Abstinence reverses EEG-indexed attention bias between drug-related and pleasant stimuli in cocaine-addicted individuals. *J. Psychiatry Neurosci.* 42 78–86. 10.1503/jpn.150358 28245173 PMC5373704

[B63] Petry N. M. Peirce J. M. Stitzer M. L. Blaine J. Roll J. M. Cohen A. (2005). Effect of prize-based incentives on outcomes in stimulant abusers in outpatient psychosocial treatment programs: A national drug abuse treatment clinical trials network study. *Arch. Gen. Psychiatry* 62 1148–1156. 10.1001/archpsyc.62.10.1148 16203960

[B64] Portrait of American Healthcare (2022). *National healthcare quality and disparities report.* Rockville, MD: Agency for Healthcare Research and Quality.

[B65] Qiu L. Nho Y. Seilheimer R. L. Kim M. J. Tufanoglu A. Williams N. (2024). Localizing electrophysiologic cue-reactivity within the nucleus accumbens guides deep brain stimulation for opioid use disorder. *bioRxiv [Preprint]* 10.1101/2024.12.30.630822 39803486 PMC11722221

[B66] Regier P. S. Jagannathan K. Franklin T. R. Wetherill R. R. Langleben D. D. Gawyrsiak M. (2021). Sustained brain response to repeated drug cues is associated with poor drug-use outcomes. *Addict. Biol.* 26:e13028. 10.1111/adb.13028 33634928 PMC9906797

[B67] Rezai A. R. Mahoney J. J. Ranjan M. Haut M. W. Zheng W. Lander L. R. (2024). Safety and feasibility clinical trial of nucleus accumbens deep brain stimulation for treatment-refractory opioid use disorder. *J. Neurosurg.* 140 231–239. 10.3171/2023.4.JNS23114 37329519

[B68] Rezai A. Thompson-Lake D. G. Y. D’Haese P. F. Meyer N. Ranjan M. Farmer D. (2025). Focused ultrasound neuromodulation: Exploring a novel treatment for severe opioid use disorder. *Biol. Psychiatry* 98 56–64. 10.1016/j.biopsych.2025.01.001 39798597 PMC12167148

[B69] Ritchie H. Arriagada P. Roser M. (2022). *Opioids, cocaine, cannabis and other illicit drugs. Our World in Data.* Available online at: https://ourworldindata.org/illicit-drug-use (accessed December 19, 2023).

[B70] Sarica C. Conner C. R. Yamamoto K. Yang A. Germann J. Lannon M. M. (2023). Trends and disparities in deep brain stimulation utilization in the United States: A nationwide inpatient sample analysis from 1993 to 2017. *Lancet Reg. Health Am.* 26:100599. 10.1016/j.lana.2023.100599 37876670 PMC10593574

[B71] Shahbabaie A. Golesorkhi M. Zamanian B. Ebrahimpoor M. Keshvari F. Nejati V. (2014). State dependent effect of transcranial direct current stimulation (tDCS) on methamphetamine craving. *Int. J. Neuropsychopharmacol.* 17 1591–1598. 10.1017/S1461145714000686 24825251

[B72] Shariatirad S. Vaziri A. Hassani-Abharian P. Sharifi Fardshad M. Molavi N. Fitzgerald P. B. (2016). Cumulative and booster effects of tdcs sessions on drug cravings, lapse, and cognitive impairment in methamphetamine use disorder: A case study report. *Am. J. Addict.* 25 264–266. 10.1111/ajad.12373 27219624

[B73] Sokhadze E. Shaban M. (2022). Event-related theta and gamma oscillations in cue-reactivity test in individuals with opiate use disorder in Buprenorphine-Maintenance program. *NeuroRegulation* 9 16–28. 10.15540/nr.9.1.16

[B74] Soyka M. Franke A. G. (2021). Recent advances in the treatment of opioid use disorders-focus on long-acting buprenorphine formulations. *World J. Psychiatry* 11 543–552. 10.5498/wjp.v11.i9.543 34631459 PMC8474991

[B75] Stagg C. J. Nitsche M. A. (2011). Physiological basis of transcranial direct current stimulation. *Neuroscientist* 17 37–53. 10.1177/1073858410386614 21343407

[B76] Su H. Chen T. Jiang H. Zhong N. Du J. Xiao K. (2020a). Intermittent theta burst transcranial magnetic stimulation for methamphetamine addiction: A randomized clinical trial. *Eur. Neuropsychopharmacol.* 31 158–161. 10.1016/j.euroneuro.2019.12.114 31902567

[B77] Su H. Liu Y. Yin D. Chen T. Li X. Zhong N. (2020b). Neuroplastic changes in resting-state functional connectivity after rTMS intervention for methamphetamine craving. *Neuropharmacology* 175:108177. 10.1016/j.neuropharm.2020.108177 32505485

[B78] Su H. Zhong N. Gan H. Wang J. Han H. Chen T. (2017). High frequency repetitive transcranial magnetic stimulation of the left dorsolateral prefrontal cortex for methamphetamine use disorders: A randomised clinical trial. *Drug Alcohol. Depend.* 175 84–91. 10.1016/j.drugalcdep.2017.01.037 28410525

[B79] Valencia-Alfonso C. E. Luigjes J. Smolders R. Cohen M. X. Levar N. Mazaheri A. (2012). Effective deep brain stimulation in heroin addiction: A case report with complementary intracranial electroencephalogram. *Biol. Psychiatry* 71 e35–e37. 10.1016/j.biopsych.2011.12.013 22281120

[B80] Vassoler F. M. Schmidt H. D. Gerard M. E. Famous K. R. Ciraulo D. A. Kornetsky C. (2008). Deep brain stimulation of the nucleus accumbens shell attenuates cocaine priming-induced reinstatement of drug seeking in rats. *J. Neurosci.* 28 8735–8739. 10.1523/JNEUROSCI.5277-07.2008 18753374 PMC2585378

[B81] Wang Y. Shen Y. Cao X. Shan C. Pan J. He H. (2016). Transcranial direct current stimulation of the frontal-parietal-temporal area attenuates cue-induced craving for heroin. *J. Psychiatry Res.* 79 1–3. 10.1016/j.jpsychires.2016.04.001 27115508

[B82] Wu H. Miller K. J. Blumenfeld Z. Williams N. R. Ravikumar V. K. Lee K. E. (2018). Closing the loop on impulsivity via nucleus accumbens delta-band activity in mice and man. *Proc. Natl. Acad. Sci. U. S. A.* 115 192–197. 10.1073/pnas.1712214114 29255043 PMC5776799

[B83] Yalachkov Y. Kaiser J. Naumer M. J. (2012). Functional neuroimaging studies in addiction: Multisensory drug stimuli and neural cue reactivity. *Neurosci. Biobehav. Rev.* 36 825–835. 10.1016/j.neubiorev.2011.12.004 22198678

[B84] Yuan J. Liu W. Liang Q. Cao X. Lucas M. V. Yuan T. F. (2020). Effect of low-frequency repetitive transcranial magnetic stimulation on impulse inhibition in abstinent patients with methamphetamine addiction: A randomized clinical trial. *JAMA Netw. Open* 3:e200910. 10.1001/jamanetworkopen.2020.0910 32167568 PMC7070234

[B85] Zammit Dimech D. Zammit Dimech A. A. Hughes M. Zrinzo L. (2024). A systematic review of deep brain stimulation for substance use disorders. *Transl. Psychiatry* 14:361. 10.1038/s41398-024-03060-1 39237552 PMC11377568

[B86] Zangen A. Moshe H. Martinez D. Barnea-Ygael N. Vapnik T. Bystritsky A. (2021). Repetitive transcranial magnetic stimulation for smoking cessation: A pivotal multicenter double-blind randomized controlled trial. *World Psychiatry* 20 397–404. 10.1002/wps.20905 34505368 PMC8429333

[B87] Zhang C. Wei H. Zhang Y. Du J. Liu W. Zhan S. (2019). Increased dopamine transporter levels following nucleus accumbens deep brain stimulation in methamphetamine use disorder: A case report. *Brain Stimul.* 12 1055–1057. 10.1016/j.brs.2019.02.023 30853339

[B88] Zhou H. Xu J. Jiang J. (2011). Deep brain stimulation of nucleus accumbens on heroin-seeking behaviors: A case report. *Biol. Psychiatry* 69 e41–e42. 10.1016/j.biopsych.2011.02.012 21489407

